# A systematic review of undisplaced femoral neck fracture treatments for patients over 65 years of age, with a focus on union rates and avascular necrosis

**DOI:** 10.1186/s13018-017-0528-9

**Published:** 2017-02-10

**Authors:** Dan-Feng Xu, Fang-Gang Bi, Chi-Yuan Ma, Zheng-Fa Wen, Xun-Zi Cai

**Affiliations:** 1 Department of Orthopaedic Surgery, The Second Affiliated Hospital, School of Medicine, Zhejiang University, Jie-fang Road 88, Hangzhou, 310009 People’s Republic of China; 2 Department of Orthopaedic Surgery, The First Affiliated Hospital of Zhengzhou University, Zhengzhou, 450001 People’s Republic of China; 3 Department of Orthopaedic Surgery, The Fourth Affiliated Hospital, School of Medicine, Zhejiang University, Yiwu, 322000 People’s Republic of China

**Keywords:** Undisplaced femoral neck fracture, Surgical treatment, Conservative treatment, Secondary displacement, Non-union, Avascular necrosis

## Abstract

**Background:**

It remains unclear whether conservative treatment should be used to treat the common undisplaced femoral neck fractures that develop in the elderly. Herein, we systematically review the rates of union and avascular necrosis after conservative and surgical treatment of undisplaced femoral neck fractures.

**Methods:**

We searched the EMBASE, PubMed, OVID, Cochrane Library, Web of Science, and Scopus databases for randomized controlled trials or observational studies that assessed the outcomes of conservative or surgical treatments of undisplaced femoral neck fractures. No language or publication year limitation was imposed. Statistical analyses were performed with the aid of the chi-squared test. We evaluated the quality of each publication and the risk of bias.

**Results:**

Twenty-nine studies involving 5071 patients were ultimately included; 1120 patients were treated conservatively and 3951 surgically. The union rates were 68.8% (642/933) and 92.6% (635/686) in the former and latter groups, respectively (*p* < 0.001). The avascular necrosis rate in the conservatively treated group was 10.3% (39/380), while it was 7.7% (159/2074) in the surgically treated group (*p* = 0.09).

**Conclusions:**

Surgery to treat undisplaced femoral neck fractures was associated with a higher union rate and a tendency toward less avascular necrosis than conservative treatment.

## Background

A femoral neck fracture (FNF) is one of the most common and devastating injuries encountered by orthopedic surgeons. Over 150,000 femoral neck fractures occur every year in the USA, and this number will double by 2050 [1–3]. In the Garden classification, Garden I and II fractures describe undisplaced FNFs in older patients [4–6]. The treatment options are conservative (bed rest with or without traction) and surgical (internal fixation) [7]. Surgical treatment was reported to be optimal [8]. However, any surgery is associated with some risk. Patients undergoing conservative treatment enjoyed good outcomes in some studies [9].

Taha et al. found that conservative therapy afforded an undisplaced FNF union rate of only 44.3% [10]. Ma et al. and Buord et al. reported that the secondary displacement rates during conservative therapy were 41 and 33%, respectively [11, 12]. However, Raaymakers et al. found that conservative treatment was successful in 85.9% of patients [9]. Surgery also seemed to be a good option, reducing secondary displacement and the non-union rate. Phillips et al. found that the union rate after surgery was 94.4% [13]. Chen et al. reported a union rate of 94.6% [14]. However, up to 22.5% of patients experienced avascular femoral head necrosis after surgery, and a fixation failure problem was also apparent [13].

Several retrospective studies have compared surgery and conservative therapy to treat undisplaced FNF. The three studies of Bentley et al., Manninger et al., and Cserhati et al. recommended surgical treatment of undisplaced FNFs; this was associated with earlier rehabilitation, lower complication rates, and higher functional scores [15–17]. However, of a total of 54 undisplaced FNF patients, Helbig et al. found that 24 (44%) developed no complications at all during conservative treatment whereas 28 (52%) required surgery because of early fracture dislocation [18]. No difference between conservative and operative treatment was evident in terms of survival rate, outcome score, or patient satisfaction.

The purpose of this systematic review was to assess all available clinical data on outcomes after surgery and conservative therapy to treat undisplaced FNFs; we mainly focused on the rates of bone union, secondary displacement, and avascular necrosis (AVN).

## Methods

### Literature search

The following sources of data were searched up to 1 October 2016 by three reviewers (DFX, CHZ, CHM): EMBASE, PubMed, OVID, Cochrane Library, Web of Science, Scopus, using the search strategy of (((femoral neck fracture [MeSH Terms]) OR (femoral neck fracture [Title/Abstract])) AND (“Garden I” OR “Garden II” OR “undisplaced” OR “non-displaced”)) with no limitation on the year or language of publication. Bibliographies of all the retrieved articles were hand-searched. In addition, we searched Clinical Trial Registry, Current Controlled Trials, Trials Central, Centre Watch, Google Scholar, multiple Websites of orthopedic associations, and conference proceedings for gray literatures. In the papers, we reviewed the references for any other papers we may not have found.

### Selection criteria

The inclusion criteria for the studies were (1) patients with an undisplaced (Garden type I or Garden type II) femoral neck fracture; (2) primarily conservative treatment; (3) primarily surgical treatment; (4) the outcomes reported at least one of the following assessments: time to union, time to weight bearing, secondary displacement, non-union, AVN, and other complications; and (5) RCTs, non-randomized or quasi-randomized controlled trials, prospective cohort trials, or retrospective comparative studies.

The exclusion criteria were (1) displaced femoral neck fracture; (2) case report, reviews, biomechanical, animal study; (3) patients and fracture that had previously been reported; (4) follow-up <6 months; and (5) sample size of <10.

### Data abstraction and analysis

Three reviews (DFX, FGB, CYM) extracted relevant data and checked the accuracy independently. Specially, study design and level of evidence, patient demographics (sample size, age, gender), mean follow-up time, loss to follow-up rate, intervention (technique and treatment protocol), and outcome measurements were all abstracted. The authors of the included trials were written to identify duplicate publication and uncertain data if necessary.

The weighted kappa for the agreement on the study quality between the investigators was 0.85 (95% confidence interval (CI), 0.77–0.93).

### Assessment of trial quality

Two reviewers (DFX and ZFW) independently assessed the methodological quality of each trial with the modified Critical Appraisal Skills Programme (CASP) [19]. Each trial was scored with 12 questions, for which the score was 1 for “Yes” and 0 for “No” or “Can’t tell”. Disagreement was evaluated by means of kappa (*κ*) test and resolved by discussion.

### Statistical analysis

All the results were combined and present as the mean value. The rates of union, secondary displacement and non-union, avascular necrosis, bed rest-related complications, 1 year mortality and reoperation could be combined for statistical analysis. Chi-square test and Fisher’s exact test were used to detect the difference of the latter two indices between the two treatment groups. The cutoff value of statistically significant difference was adjusted as *α’* = *α*/[(*k*/2) + 1] = 0.05/[(4/2) + 1] = 0.017, where *k* was the number of groups. The pooling of the functional assessment data sets was not attempted because of the significant variability in the criteria. Statistical analyses were performed through STATA 12.0 (Stata Corp., College Station, TX, USA).

## Results

### Study identification

**Fig. 1:**
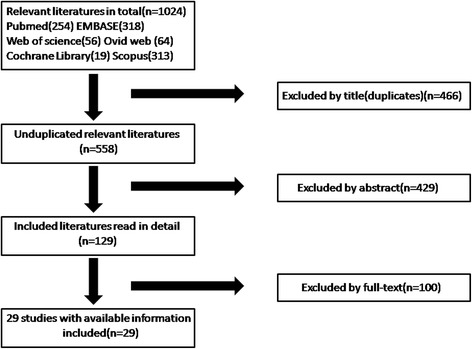
A PRISMA flowchart illustrated the selection of studies included in our systematic review

The initial literature search yielded 1024 articles including 466 duplicates, after the removal of which 558 articles remained. Of these, 429 were excluded because they did not fulfill our selection or exclusion criteria based on evaluation of the titles and abstracts. The full texts of the remaining 129 papers were reviewed, and 31 [9–15, 17, 20–28] (Fig. 1) were finally included; these dealt with patients with undisplaced FNFs who were managed either non-operatively or operatively. After careful inspection, we found that >50% of the patients were lost to follow-up in the study of Manninger et al. [16] Additionally, in another study, patients in the surgical group had initially received conservative therapy [18]. We excluded both studies; 29 studies thus remained.

### Patient characteristics and interventions

Table 1 shows the patient characteristics and the interventions used in all trials. There were 5 prospective cohort studies [9, 12, 23, 27, 29] and 24 retrospective cohort studies [10–15, 17, 20–22, 24–26, 28, 30–40]. Of the 29 studies, 2 [15, 17] compared both therapies, 7 [9–12, 26–28] explored the outcomes of conservative treatment, and 20 [13, 14, 20–25, 29–40] the outcomes of surgery. The surgical options included the use of cancellous screws, single Watson-Jones nails, placement of three Knowles pins, use of a dynamic hip screw, and Smith-Petersen nails. All trials recruited >10 patients. In total, 5071 cases were included, of which 1120 were conservatively and 3951 surgically treated. The vast majority of patients were female (female/male = 3211/1280). Four papers did not indicate the gender distributions [27, 32, 35, 39]. The mean follow-up duration was >28.3 months. The frequency-weighted mean age was 75.0 ± 4.8 years for the conservatively treated group and 76.5 ± 4.1 years for the surgically treated group.

**Table 1:** Study characteristics and intervention of the trials with operation or non-operation or comparison

Study	Level	Patients/cases	Mean age (years)	F/M	Follow-up (months)	Garden I/Garden II	Treatment
Comparison studies							
Bentley [1]	IV	43/43	72	58/8	>36	NR	Conservation
		23/23			>36	NR	20 Smith-Petersen nailing, 2 three Moore pins, 1 Charnley compression screw
Cserhati [2]	IV	122/122	74.6	98/24	NR	106/16	Conservation
		125/125	73.3	104/21	NR	98/27	19 two Smith-Petersen nail, 5 three cannulated screws, 95 three screws + key-hole plage, 6 others
Conservative treatment studies							
Ma [3]	IV	115/115	71	88/27	NR	115/0	
Jensen [4]	IV	128/128	73	101/27	22	85/43	
Buorda [5]	III	57/57	81.8	50/7	20	NR	
Taha [6]	IV	61/61	83	48/13	NR	NR	
Raaymakers [7]	III	170/170	69.8	130/40	NR	167/0	
Raaymakers [8]	III	319/319	72	NR	NR	311/0	
Verheyen [9]	IV	105/105	78	83/22	NR	105/0	
Operative treatment studies							
Rogmark [10]	IV	224/224	81	156/68	32	NR	Two Hansson hook pins
Lapidus [11]	III	382/382	80.7	282/100	79.2	NR	Olmed screws
Han [12]	IV	52/52	77.6	38/14	11.7	13/39	Multiple screws, pins, or dynamic hip screw
Phillips [13]	IV	100/100	76.9	86/14	22.6	NR	Watson-Jones nail fixation
Manohara [14]	IV	100/100	78	77/23	39	85/15	Screws
Kim [15]	IV	60/60	77.5	40/18	46.8	28/30	Three cannulated screws
Chiu [16]	IV	298/305	66	197/101	75	NR	Knowles pins
Chen [17]	IV	37/37	83.7	22/15	>24	21/16	Cancellous screws
Makki [18]	IV	31/31	75	22/9	>12	2/26	Dynamic hip screw
		34/34	70	20/14	>12	9/25	Dynamic hip screw with anti-rotation screw
Parker [19]	IV	346/346	80.8	295/51	>12	NR	Screw
		346/346	80.8	295/51	>12	NR	Hemiarthroplasty
Stromqvist [20]	IV	85/85	78	NR	>12	NR	Hook Pin
Parker [21]	IV	565/565	76	435/130	>12	NR	A sliding hip screw or three cannulated screws
Parker [22]	IV	112/112	76	NR	24	NR	Internal fixation
Lee [23]	IV	28/28	70.1	18/15	>12	NR	Dynamic hip screw
		22/22	74.6	13/12	>12	NR	Dynamic hip screw
		27/27	72.8	15/17	>12	NR	Multiple cannulated screws
Hui [24]	IV	15/15	>80	NR	>6	NR	Over 80 years internal fixation
		16/16	72	NR	>6	NR	65–79 years internal fixation
		23/23	>80	NR	>6	NR	Over 80 years hemiarthroplasty
Bjorgul [25]	IV	225/225	80	161/64	47	NR	Two cannulated screws
Watson [26]	III	31/31	77.9	25/6	>24	NR	Dynamic hip screw
		29/29	76.7	24/5	>24	NR	Three partially threaded screws
Yih [27]	IV	46/46	72.8	24/16	33.5	NR	Dynamic hip screws
		48/48	70.6	25/19	35.6	NR	Multiple cannulated screws
Conn [28]	IV	375/375	77.1	75/296	>12	NR	Three parallel
Sikand [29]	IV	29/29	79	21/8	>12	NR	Hemiarthroplasty
		110/110	77	85/25	>12	NR	Internal fixation

*NR* not recorded

### Outcomes

Tables 2 and 3 show the outcome measurements of all trials. All trials reported secondary displacement and/or non-union rates, and those of later AVN. Fourteen studies [9–11, 13–15, 20, 25–28, 33, 34, 40] reported union rates. These were 68.8% (642/933) in those receiving conservative treatment and 92.6% (635/686) in those receiving surgical treatment. The secondary displacement rate was 30.0% (334/1112) in conservatively treated patients compared to 0.57% (12/2124) in surgically treated patients. The AVN rate was 10.3% (39/380) in conservatively treated patients compared with 7.7% (159/2074) in surgically treated patients. The re-operation rate was 22.6% (157/696) in conservatively treated patients compared with 10.6% (336/3155) in surgically treated patients. Bed rest-related complications developed in 11.4% (27/237) of conservatively treated patients compared with 4.9% (106/2149) of surgically treated ones. The 1-year mortality was 14.7% (125/852) in conservatively treated patients compared with 18% (598/3318) in surgically treated ones.

**Table 2:** Details of outcome measurements of the trials

Study	Treatment	Union	Secondary displacement	Non-union	Fixation failure	Avascular necrosis	Bed rest-related complications	1 year mortality	Re-operation	Reduction in mobility status	Local pain
Comparison studies											
Bentley [1]	Conservation	83.7%(36/43)	16.3%(7/43)	2.3%(1/43)	NR	14.0%(6/43)	NR	NR	14%(5/43)	NR	NR
	Petersen nailing, Moore pins	95.7%(22/23)	0(0/23)	4.3%(1/23)	4.3%(1/23)	17.4%(4/23)	NR	NR	0(0/23)	NR	NR
Cserhati [2]	Conservation	NR	19.7%(24/122)	0.8%(1/122)	NR	12.8%(5/39)	18.9%(23/122)	13.9%(17/122)	18%(22/122)	25.6%(10/39)	51.3%(20/39)
	Petersen nail, three screws + key-hole plage	NR	0.8%(1/125)	0(0/125)	0.8%(1/125)	16.1%(10/62)	3.2%(4/125)	10.4%(13/125)	NR	45.2(28/62)	61.3%(38/62)
Conservative treatment studies											
Jensen [3]	Conservation	72.7%(93/128)	27.3%(35/128)	NR	NR	NR	NR	NR	NR	NR	NR
Ma [4]	Conservation	58.3%(67/115)	41.7%(48/115)	NR	NR	NR	3.5%(4/115)	3.5%(4/115)	41.7%(48/115)	NR	NR
Raaymakers [5]	Conservation	69%(216/311)	31%(95/311)	NR	NR	11.3%(18/160)	NR	19.1%(61/319)	10.6%(33/311)	NR	NR
Raaymakers [6]	Conservation	85.9%(146/170)	14.1%(24/170)	NR	NR	11.1%(9/81)	NR	16.5%(28/170)	NR	NR	NR
Verheyen [7]	Conservation	54.3%(57/105)	45.7%(48/105)	NR	NR	NR	NR	NR	45.7%(48/105)	NR	NR
Buorda [8]	Conservation	NR	33.3%(19/57)	NR	NR	1.8%(1/57)	NR	29.8%(17/57)	NR	NR	NR
Taha [9]	Conservation	44.3%(27/61)	55.7%(34/61)	NR	NR	NR	NR	4.9%(3/61)	NR	NR	NR
Operative treatment studies											
Kim [10]	Three cannulated screws	NR	0(0/58)	0(0/58)	1.9%(1/54)	7.4%(4/54)	NR	6.9%(4/58)	7.4%(4/54)	35.2%(19/54)	NR
Manohara [11]	Cancellous screws	NR	0(0/100)	0(0/100)	3%(3/100)	5%(5/100)	7%(7/100)	18%(18/100)	8%(8/100)	44.1%(30/68)	22.1%(15/68)
Han [12]	Multiple screws, pins, or dynamic hip screw	NR	0(0/52)	15.4%(8/52)	NR	11.5%(6/52)	1.9%(1/52)	3.8%(2/52)	30.8%(16/52)	NR	NR
Phillips [13]	Watson-Jones nail	94.4%(68/72)	0(0/72)	5.6%(4/72)	5.6%(4/72)	22.5%(9/40)	NR	29.2%(21/72)	9.7%(7/72)	NR	NR
Lapidus [14]	Olmed screws	NR	0(0/382)	6.5%(25/382)	NR	5.2%(20/382)	4.5%(17/382)	21.5%(82/382)	11.8%(45/382)	NR	5%(19/382)
Rogmark [15]	Hansson hook pins	NR	0(0/224)	4.5%(10/224)	7.1%(16/224)	5.4%(12/224)	11.2%(25/224)	11.2%(25/224)	15.2%(34/224)	70.6%(72/102)	36.4%(36/99)
Chen [16]	Cancellous screws	94.6% (35/37)	5.4% (2/37)	0(0/37)	NR	10.8% (4/37)	NR	NR	16.2%(6/37)	NR	51.4(19/37)
Chiu [17]	Knowles pins	93.4%(285/305)	0(0/305)	5.6%(17/305)	3.0%(9/305)	7.2%(22/305)	NR	13.7%(50/366)	12.8%(39/305)	NR	7.2%(22/305)
Makki [18]	Dynamic hip screw	96.8%(30/31)	NR	3.2%(1/31)	NR	3.2%(1/31)	NR	9.7%(3/31)	0(0/31)	NR	NR
	Dynamic hip screw with anti-rotation screw	88.2%(30/34)	NR	11.8%(4/34)	NR	8.8%(3/34)	NR	2.9%(1/34)	2.9%(1/34)	NR	NR
Parker [19]	Screw fixation	NR	0(0/346)	11%(38/346)	NR	NR	1.7%(6/346)	18.8%(65/346)	14.5%(50/346)	33.2%(115/346)	NR
	Hemiarthroplasty	NR	0.58%(2/346)	3.8%(13/346)	NR	NR	6.9%(24/346)	25.7%(89/346)	5.5%(19/346)	19.1%(66/346)	NR
Stromqvist [20]	Hook pin fixation	NR	NR	1.6%(1/64)	NR	3.1%(2/64)	NR	24.7%(21/85)	NR	NR	NR
Parker [21]	Three cannulated screws	NR	NR	8.5%(48/565)	NR	NR	NR	NR	NR	NR	NR
Parker [22]	Internal fixation	NR	NR	2.7%(3/112)	NR	4.5%(5/112)	NR	NR	4.5%(5/112)	NR	NR
Yih [23]	Minimally invasive dynamic hip screw	100%(33/33)	NR	0(0/33)	0(0/33)	12.1%(4/33)	NR	9.1%(3/33)	3%(1/33)	NR	NR
	Dynamic hip screw	100%(25/25)	NR	0(0/25)	0(0/25)	12%(3/25)	NR	8%(2/25)	4%(1/25)	NR	NR
	Multiple cannulated screws	96.9%(31/32)	NR	3.1%(1/32)	9.4%(3/32)	21.9%(7/32)	NR	9.4%(3/32)	15.6%(5/32)	NR	NR
Hui [24]	Over 80 years internal fixation	NR	26.7%(4/15)	NR	6.7%(1/15)	26.7%(4/15)	NR	17.2%(5/29)	53.3%(8/15)	NR	6.7%(1/15)
	65 to 79 years internal fixation	NR	6.3%(1/16)	NR	0(0/16)	0(0/16)	NR	25%(7/28)	12.5%(2/16)	NR	12.5%(2/16)
	Over 80 years hemiarthroplasty	NR	8.7%(2/23)	NR	0(0/23)	0(0/23)	NR	33.3%(19/57)	13%(3/23)	4.3%(1/23)	4.3%(1/23)
Bjorgul [25]	Cannulated screws	NR	1.8%(4/225)	7.1%(16/225)	1.3%(3/225)	4.4%(10/225)	NR	14.7%(33/225)	18.7%(42/225)	36.9%(83/225)	2.7%(6/225)
Watson [26]	Dynamic hip screw	NR	NR	NR	NR	3.2%(1/31)	9.7%(3/31)	19.4%(6/31)	3.2%(1/31)	32.3%(10/31)	32.3%(10/31)
	Cancellous screws.	NR	NR	NR	NR	0(0/29)	6.9%(2/29)	20.7%(6/29)	10.3%(3/29)	31%(9/29)	20.7%(6/29)
Lee [27]	Dynamic hip screws	84.8%(39/46)	NR	0(0/46)	0(0/46)	8.7%(4/46)	NR	6.5%(3/46)	2.2%(1/46)	NR	NR
	Multiple cannulated screws	77%(37/48)	NR	4.2%(2/48)	6.3%(3/48)	8.3%(4/48)	NR	4.2%(2/48)	14.6%(7/48)	NR	NR
Conn [28]	Three parallel 6.5-mm AO	NR	NR	6.4%(24/375)	NR	4%(15/375)	3.7%(14/375)	20.5%(77/375)	5.1%(19/375)	NR	NR
Sikand [29]	Conservation	NR	NR	NR	NR	NR	NR	50%(4/8)	NR	NR	NR
	Hemiarthroplasty	NR	NR	NR	NR	NR	6.9%(2/29)	37.9%(11/29)	3.4%(1/29)	37.9%(11/29)	NR
	Internal fixation	NR	NR	NR	NR	NR	0.9%(1/110)	15.5%(17/110)	7.3%(8/110)	39.1%(43/110)	NR

*NR* not record

Bed rest-related complications: deep venous thrombosis, pneumonia, and urinary infection

**Table 3:** Demographics according to treatment group

Variable	Conservative treatment	Surgical treatment	*P* value
Cases	1120	3951	
Mean age (years)	75.0 ± 4.8	76.5 ± 4.1	
Female/Male	598/160	2555/1112	
Union	68.8%(642/933)	92.6%(635/686)	<0.001
Secondary displacement and non-union	30%(334/1112)	0.57%(12/2124)	<0.001
Avascular necrosis	10.3%(39/380)	7.7%(159/2074)	0.09
Bed rest-related complications	11.4%(27/237)	4.9%(106/2149)	<0.001
1 year mortality	14.7%(125/852)	18%(598/3318)	0.02
Reoperation	22.6%(157/696)	10.6%(336/3155)	<0.001

### Literature quality and the risk of bias

Most studies scored moderately in terms of methodological quality. The overall score was 7.38 ± 1.37, rendering the outcomes susceptible to the risk of bias (Table 4). The weighted kappa for agreement on trial quality between reviewers was 0.83 [95% CI (0.72–0.92)]. The biases included:

**Table 4:** Methodological quality of the included studies based on the modified CASP

Literatures	A focused issue with a question	Method appropriate	Patients recruited appropiately	Define the intervention	Outcomes accurately measured	Confounding factors identified	Confounding factors considered	Follow-up >80%	Results all clearly defined	Results realistic?	Applicable to local population	Results proportional to other evidence	Total
Bentley [1]	1	1	C.T	1	1	0	1	1	1	1	1	1	10
Cserhati [2]	1	1	0	1	1	0	0	0	0	1	1	1	7
Ma [3]	1	0	0	0	0	0	0	1	0	1	1	1	5
Jensen [4]	1	0	0	0	1	0	1	1	0	1	1	1	7
Buorda [5]	1	0	1	0	1	0	1	1	1	1	1	1	9
Taha [6]	1	0	C.T	0	0	0	0	1	0	1	1	1	5
Raaymakers [7]	1	0	C.T	0	0	0	1	1	1	1	1	1	7
Raaymakers [8]	1	0	C.T	0	0	0	1	1	1	1	1	1	7
Verheyen [9]	1	0	0	0	0	0	1	1	0	1	1	1	6
Rogmark [10]	1	0	0	0	1	0	1	1	0	1	1	1	7
Lapidus [11]	1	0	C.T	0	0	0	1	1	1	1	1	1	7
Han [12]	1	0	0	0	0	0	1	1	1	1	1	1	7
Phillips [13]	1	0	1	0	0	0	1	0	1	1	1	1	7
Manohara [14]	1	0	0	0	1	0	1	1	1	1	1	1	8
Kim [15]	1	0	1	0	1	0	1	1	1	1	1	1	9
Chiu [16]	1	0	1	0	1	0	1	1	0	1	1	1	8
Chen [17]	1	0	C.T	0	0	0	1	1	1	1	1	1	7
Makki [18]	1	0	0	0	1	0	1	1	1	1	1	1	8
Parker [19]	1	0	1	0	1	0	1	1	1	1	1	1	9
Stromqvist [20]	1	0	0	0	1	0	0	1	0	1	1	1	6
Parker [21]	1	0	1	0	1	0	1	1	1	1	1	1	9
Parker [22]	1	0	1	0	1	0	0	1	0	1	1	1	7
Lee [23]	1	0	1	0	1	0	1	1	1	1	1	1	9
Hui [24]	1	0	C.T	0	0	0	0	1	0	1	1	1	5
Bjorgul [25]	1	0	1	0	1	0	1	1	0	1	1	1	8
Watson [26]	1	0	1	0	1	0	1	1	1	1	1	1	9
Yih [27]	1	0	0	0	1	0	0	1	0	1	1	1	6
Conn [28]	1	0	1	0	1	0	1	1	1	1	1	1	9
Sikand [29]	1	0	0	0	0	0	1	1	0	1	1	1	6
Mean	1.00	0.07	0.38	0.07	0.62	0.00	0.76	0.93	0.55	1	1.00	1.00	7.38

*C.T* cannot tell

1. Selection bias: The fact that few trials were typical RCTs may cause major selection bias. Inconsistencies in evaluation of the type of undisplaced FNF and patient age may constitute other sources of bias.
2. Performance bias: This is attributable to the lack of rehabilitation programs. No consistent method was used for early weight-bearing facilitating recovery.
3. Attrition bias: A small number of trials exhibited considerable loss to follow-up. Most studies reported outcomes incompletely.
4. Detection bias: This may possibly be caused by non-standardized or undescribed follow-up schedules.
5. Reporting bias: This is an intrinsic weakness of retrospective cohort studies.

In detail, we restricted the type of FNF to undisplaced fractures. Most studies contained patients with both undisplaced and displaced fractures. It was difficult to isolate data on undisplaced FNF; we therefore established strict selection and exclusion criteria to reduce selection bias as much as possible. Furthermore, differences in surgical methods increased performance bias. Inconsistencies in follow-up and loss to follow-up also increased bias. Most studies were retrospective in nature, rendering reporting bias unavoidable.

### Pooled analysis

As the measurements of patient characteristics and outcomes were consistent among the trials, we pooled these to derive mean values. Overall, surgically treated patients had a shorter time to weight-bearing and a shorter hospital stay. In addition, such patients had a higher union rate, lower rates of secondary displacement and non-union, and a lower rate of bed rest-related complications.

Significant differences were evident between the surgical and the conservative treatment groups in terms of the union rate (*p* < 0.001), the rates of secondary displacement and non-union (*p* < 0.001), and the rate of bed rest-related complications (*p* < 0.001). A trend toward a difference in the AVN rate was also apparent (*p* = 0.09).

## Discussion

To the best of our knowledge, this is the first systematic review to focus on the optimal treatment for undisplaced FNFs. We investigated whether conservative treatment was optimal for the common problem of undisplaced FNFs in the elderly. We developed explicit inclusion and exclusion criteria, assessed the methodological quality of all studies, explored the reproducibility of all selection and assessment criteria, performed quantitative analysis, and explored possible reasons for observed differences among studies. We found data paradox and confirmed the correct data of the original paper [20]. The validity of our findings is strengthened by the fact that we strictly followed the suggestions of the Cochrane Handbook for Systematic Reviews of Interventions (version 5.0.2) and the PRISMA 2009 checklist.

One of our most important findings is that fractures that were surgically treated had higher union rates and comparable non-union rates to those treated conservatively. Obviously, fixation affords stability and stiffness, directly enhancing the strength of the femoral neck [41, 42]. Biomechanical studies have confirmed that fracture fixation and immobilization affect the pattern of skeletogenic stem cell differentiation into osteoblasts; mechanical fixation would obviously influence neovascularization [43]. Thus, fixation promotes bone union. In some studies [13–15, 20], the union rates reached 90%. Fixation failure is a rare complication after surgery to treat undisplaced fractures. The fixation failure rate in our meta-analysis was only 3.3% (45/1366).

Conservative treatment is an option for undisplaced FNFs, the advantage being that surgery is avoided, but most studies revealed a significant risk of displacement during non-operative treatment. The risk varied from 14.1 to 55.7% [9–12, 26–28]. Verheyen et al. [28] explored the rate of secondary displacement in 105 patients. Forty-eight patients (46%) were at risk of such displacement; the patient group had a high mean age. Secondary displacement was more common in patients aged >70 years, in agreement with the data of Raaymakers [27], who reported secondary instability in 41% of patients >70 years of age. In healthy patients <70 years of age, the value was 7%. Secondary displacement is very rare after surgical treatment.

AVN is a well-recognized complication of FNFs, caused by alterations in the blood supply [7, 44]. AVN often develops 2–3 years after treatment. We found no significant difference between the two treatment groups in terms of AVN. However, AVN tended to be less common in surgical patients. Massive rupture of the retinacular vessels may occur when the femoral head is rotated during surgery; this may trigger AVN [45]. Thus, fixation potentially adversely affects the vascularity of the femoral head [46]. On the other hand, fixation prevents micromotion of the fracture site, facilitating vascularity. In Brodetti et al.’s cadaver experimental study [47], they inserted nails or screws into various sites to observe changes in blood supply; they found that such insertions were unlikely to contribute to further devascularization of the femoral head. Bentley et al. [15] followed up patients for >3 years and found that, although no AVN occurred in the first year, AVN did develop after 2 years in both groups. The incidence did not vary greatly between those who underwent conservative (14%) and surgical (18%) treatment. Hence, follow-up is very important to AVN detection.

Re-operation to deal with complications is a commonly reported outcome measure. After conservative treatment, re-operations were principally attributable to secondary displacement and latent AVN [11, 28]. Non-union, fixation failure, and AVN were the most common reasons for re-operation after surgical treatment [7]. The re-operation rate differed significantly (*p* < 0.01) between the two treatment options. Overall, the outcomes were better after surgery. It is true that patients are exposed to extra risks (including anesthesia and bleeding) during surgery. We found no evidence suggesting that complications associated with anesthesia and surgery outweighed the increased risk of fracture-healing complications characteristic of conservative treatment. On the contrary, surgical treatment significantly reduced the risk of fracture displacement and significantly increased the union rate. Patients undergoing re-operation generally underwent hemiarthroplasty or arthroplasty [7, 11]. Overall, surgical treatment must be recommended.

Many clinical reports on treatment outcomes have focused on surgical rather than functional outcome measures. The objective functional results of various treatments are rarely assessed. The Harris hip score (HHS) is the most common modality used to assess hip function. In one study [40] on patients aged >60 years, Yih et al. reported an HHS of 84.2 ± 5.2 for those treated via insertion of dynamic hip screws and 82.6 ± 5.1 for those undergoing osteosynthesis using cannulated screws. Some studies employed fairly crude outcome measures (pain and mobility level documented in a rudimentary manner). In a recent study of 224 patients who completed self-evaluation questionnaires >3 years after internal fixation, Rogmark et al. found that 40% reported average to severe pain when walking and 25% pain at rest. [25] Functional results are rarely assessed in those treated conservatively. In one comparative study [17], Cserhati et al. recorded the levels of pain and mobility. Of 39 patients, 5.6% (10/39) reported poorer mobility status after conservative treatment compared with 45.2% (28/62) of those who underwent surgery; 51.3% (20/39) of patients reported severe or moderate pain when weight-bearing after conservative treatment compared with 61.3% (38/62) of those who underwent surgical treatment. It thus seems that conservative treatment afforded better outcomes. In terms of mortality, this was higher (68%; 83/122) after conservative treatment than the 50.4% (63/125) after surgical treatment. Therefore, the overall outcome is better after surgical treatment.

In terms of surgery, primary hemiarthroplasty of an undisplaced FNF is a possible alternative treatment. Parker et al. [37] randomized 692 patients with undisplaced FNFs to hemiarthroplasty (346 patients) or internal fixation (346 patients). Fixation was associated with a significantly reduced operative time (43 vs. 67 min), less blood loss, and a lower 1-year mortality rate (19 vs. 26%). The additional benefits of fixation were less pain at 1 year, less reduction in mobility, and a reduced dependence on walking aids. Re-operations were required by 5.5% (19/346) of the hemiarthroplasty group and 14.5% (50/346) of the fixation group. Hui et al. [32] and Sikand et al. [38] also evaluated re-operation and mortality levels. Internal fixation was associated with lower mortality but a greater need for re-operation, compared with hemiarthroplasty. The increased risk of mortality associated with hemiarthroplasty indicates that hemiarthroplasty cannot be recommended to treat an undisplaced FNF.

A precise diagnosis is important prior to choosing a treatment option. Radiography has certain limitations when used to distinguish FNF types, which can result in misdiagnosis. A patient may in fact have a displaced FNF but be diagnosed with an undisplaced one [6, 10]. In addition, diagnoses using the Garden classification are very inconsistent. Zlowodzki et al. [48] surveyed the preferences of orthopedic surgeons in terms of FNF classification systems and asked whether they thought they could distinguish the four different Garden fracture types. Of all surgeons, 96% felt that they could distinguish between undisplaced (Garden I/II) and displaced (Garden III/IV) fractures. However, the Garden classification system exhibits great variability. Therefore, X-rays combined with a CT scan should be routinely used for diagnosis [6].

We reviewed the conservative treatments employed [9–12, 15, 17, 26–28]. Careful nursing and optimal physician management allowed gradual mobilization to commence with exercises in bed, followed by partial weight-bearing, with the aid of crutches, for ≥8 weeks after fracture. The outcomes were satisfactory [9, 15]. However, only a few patients adhered to their rehabilitative protocols in the long term. If a patient without a comorbid condition can guarantee good compliance, conservative treatment may also be recommended. In addition, patients with surgical risks must be treated conservatively.

Our work had certain limitations: (1) Most studies were retrospective in nature. Ignoring such studies would have underpowered our analyses and negatively affected the accuracy of our findings. A future strictly designed and adequately powered RCT is essential. (2) We pooled Garden I and Garden II FNFs; their prognoses did not differ greatly. A future study could compare treatment outcomes between patients with these two types of FNF. (3) We explored possible publication bias using Begg, Egger, and funnel plots. The included studies did not meet the standards required by these methods; it was thus difficult to evaluate publication bias.

Ideally, a randomized trial would reveal whether surgical or conservative treatment should be preferred for undisplaced FNFs. We suggest that future studies should prospectively compare the outcomes and complication rates of different internal fixation techniques and conservative methods.

## Conclusions

Surgery to treat undisplaced FNFs was associated with a higher union rate and a tendency toward reduction in the AVN rate. Careful treatment and follow-up are essential. We suggest that CT should be routinely used to assist in precise diagnosis. Follow-up should be maintained for at least 2 years, allowing AVN detection (if AVN develops) and treatment.

## Abbreviations

AVN: Avascular necrosis
FNF: Femoral neck fractures
HHS: Harris hip score
